# Investigation of vertical and horizontal transmission of *Spiroplasma* in ticks under laboratory conditions

**DOI:** 10.1038/s41598-023-39128-z

**Published:** 2023-08-15

**Authors:** Shohei Ogata, Rika Umemiya-Shirafuji, Kodai Kusakisako, Keita Kakisaka, Elisha Chatanga, Naoki Hayashi, Yurie Taya, Yuma Ohari, Gita Sadaula Pandey, Abdelbaset Eweda Abdelbaset, Yongjin Qiu, Keita Matsuno, Nariaki Nonaka, Ryo Nakao

**Affiliations:** 1 Laboratory of Parasitology, Department of Disease Control, Graduate School of Infectious Diseases, Faculty of Veterinary Medicine, Hokkaido University, Sapporo, 060-0818 Japan; 2 Laboratory of Molecular Targeted Therapeutics, School of Pharmacy, Nihon University, Chiba, 274-8555 Japan; 3 Division of International Research Promotion, International Institute for Zoonosis Control, Hokkaido University, Sapporo, 001-0020 Japan; 4 National Research Center for Protozoan Diseases, Obihiro University of Agriculture and Veterinary Medicine, Obihiro, 080-8555 Japan; 5 Laboratory of Veterinary Parasitology, School of Veterinary Medicine, Kitasato University, Towada, 034-8628 Japan; 6 Department of Veterinary Pathobiology, Faculty of Veterinary Medicine, Lilongwe University of Agriculture and Natural Resources, P.O. Box 219, Lilongwe, Malawi; 7 School of Veterinary Medicine, Rakuno Gakuen University, Ebetsu, 069-8501 Japan; 8 Department of Animal Medicine, Clinical Laboratory Diagnosis, Faculty of Veterinary Medicine, Assiut University, Assiut, 71515 Egypt; 9 Department of Virology-I, National Institute of Infectious Diseases, Shinjuku-ku, Tokyo 162-8640 Japan; 10 Management Department of Biosafety, Laboratory Animal, and Pathogen Bank, National Institute of Infectious Diseases, Shinjuku-ku, Tokyo 162-8640 Japan; 11 Division of Risk Analysis and Management, International Institute for Zoonosis Control, Hokkaido University, Sapporo, 001-0020 Japan; 12 One Health Research Center, Hokkaido University, Sapporo, 001-0020 Japan; 13 International Collaboration Unit, International Institute for Zoonosis Control, Hokkaido University, Sapporo, 001-0020 Japan

**Keywords:** Entomology, Parasitology

## Abstract

Many arthropods harbour bacterial symbionts, which are maintained by vertical and/or horizontal transmission. *Spiroplasma* is one of the most well-known symbionts of ticks and other arthropods. It is still unclear how *Spiroplasma* infections have spread in tick populations despite its high prevalence in some tick species. In this study, *Ixodes ovatus*, which has been reported to harbour *Spiroplasma ixodetis* at high frequencies, was examined for its vertical transmission potential under experimental conditions. Next, two isolates of tick-derived *Spiroplasma*, *S. ixodetis* and *Spiroplasma mirum*, were experimentally inoculated into *Spiroplasma*-free *Haemaphysalis longicornis* colonies and the presence of *Spiroplasma* in their eggs and larvae was tested. Our experimental data confirmed that *S. ixodetis* was transmitted to eggs and larvae in a vertical manner in the original host *I. ovatus*. In the second experiment, there was no significant difference in engorged weight, egg weight, and hatching rate between *Spiroplasma*-inoculated and control *H. longicornis* groups. This suggested that *Spiroplasma* infection does not affect tick reproduction. *Spiroplasma* DNA was only detected in the eggs and larvae derived from some individuals of *S. ixodetis*-inoculated groups. This has demonstrated the potential of horizontal transmission between different tick species. These findings may help understand the transmission dynamics of *Spiroplasma* in nature and its adaptation mechanism to host arthropod species.

## Introduction

Ixodid ticks are blood-feeding ectoparasites of vertebrates with approximately 700 species distributed worldwide^1^. They serve as vectors for many pathogens and cause significant global public health and veterinary problems^2,3^. Ixodid ticks attach to their hosts for days or weeks while feeding on the blood. Depending on the number of hosts that they infest during their life cycle, they are divided into one-, two-, and three-host ticks. One-host ticks attach to a single host during their life cycle, while two- and three-host ticks drop off the host after feeding to moult in the environment between stages^4–8^.

The term ‘symbiosis’ was first defined in 1879 to describe the condition where different species live together in close association^9^. Symbiotic relationships are important in biological processes and ecological systems^10^. In nature, symbiotic relationships between bacteria and arthropods are well known and have been extensively studied^10^. Symbionts use disparate transmission strategies to allow their survival in their hosts^11^. Some symbionts such as *Wolbachia* and *Arsenophonus* are transmitted vertically in host arthropods^12^. Meanwhile, others use horizontal transmission routes, for instance, by being acquired from environmental or infected conspecifics or other species^13,14^. In some cases, a combination of multiple infection routes creates complex transmission dynamics in nature^15,16^. The dominant transmission form of endosymbionts is vertical transmission, which occurs primarily from the mother to offspring^17^. Some insect symbionts display specialised transmission strategies, for instance, via parental post-oviposition secretions^18^.

Members of the genus *Spiroplasma* are helical bacteria that lack cell walls. It is estimated that 5–10% of arthropods harbour *Spiroplasma* as their symbionts^19,20^. Many species of *Spiroplasma* maintain their infection in their hosts through vertical transmission^21^. In *Drosophila*, *Spiroplasma* uses yolk uptake machinery to move into the germline for vertical transmission^22^. It has recently been found that some of these vertically transmitted *Spiroplasma* confer protection against nematodes, parasitoid wasps, and fungi to their hosts^21^. Although vertical transmission of *Spiroplasma* has been confirmed in some species^21^, phylogenetic studies have reported poor clustering of *Spiroplasma* including from the same host species. This suggests that horizontal transmission between unrelated hosts occurs frequently^21,23,24^.

Ticks generally harbour maternally inherited bacterial endosymbionts^25,26^. Some of these endosymbionts, such as *Coxiella* and *Francisella* are likely essential for the life cycle of ticks^27^. Ticks have also been shown to harbour *Spiroplasma*^28,29^*.* How *Spiroplasma* infection spreads among tick populations is still unclear despite high infection rates being observed in some tick species, such as *Ixodes ovatus* and *Haemaphysalis kitaokai*^30^.

In this study, the vertical transmission of *Spiroplasma* was demonstrated using field-collected *I. ovatus*, which harbours *Spiroplasma* at high frequencies. In addition, the horizontal transmission potential of *Spiroplasma* was examined by experimentally inoculating *Spiroplasma* strains isolated from ticks into *Spiroplasma*-free laboratory tick colonies.

## Results

### Detection of *Spiroplasma* in eggs and larvae of *I. ovatus*

Out of 30 *I. ovatus* females (mated beforehand with males) used to infest rabbits, 14 ticks became fully engorged and detached from the animals eight days after infestation. Nine of them laid eggs approximately nine days after detachment, while oviposition was not observed in five engorged ticks.

DNA extracted from three pools of eggs and three pools of larvae per tick were used for detection of *Spiroplasma* by PCR and Sanger sequencing. *Spiroplasma ixodetis* DNA was detected in all the egg and larval pools tested. *Spiroplasma* infection was also confirmed in all the engorged *I. ovatus* females from which the eggs and larvae tested were obtained.

### Experimental inoculation of *Spiroplasma* into *H. longicornis*

**Figure 1:**
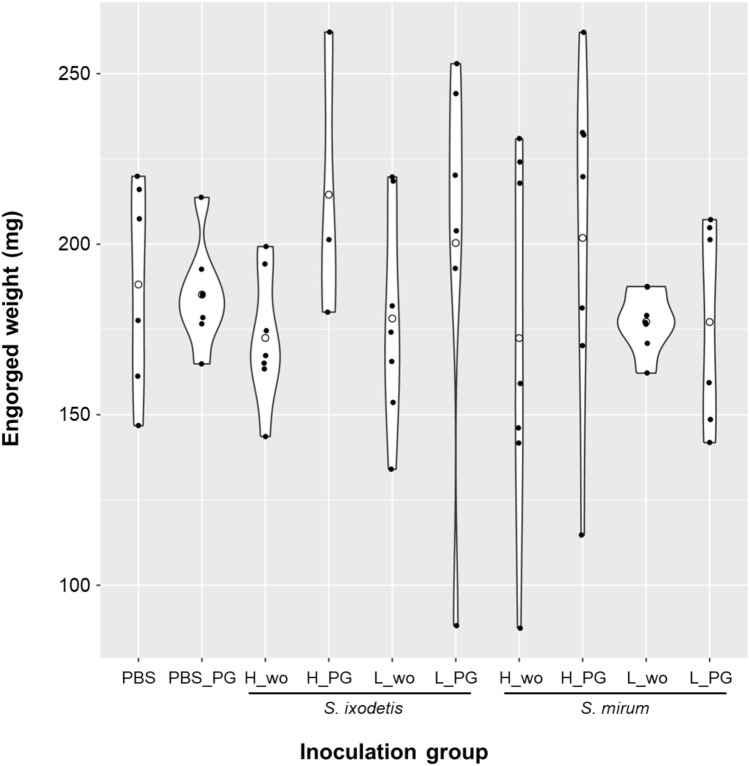
Engorged weight of ticks after the experimental inoculations. PBS_PG is PBS + penicillin G sodium salt. H_wo is 5 × 10^11^ bacteria. H_PG is 5 × 10^11^ bacteria + penicillin G sodium salt. L_wo is 5 × 10^8^ bacteria. L_PG is 5 × 10^8^ bacteria + penicillin G sodium salt. The white circle in the figure represents the average engorged weight. The black circle in the figure represents each value.

Females of *H. longicornis* were injected with either PBS, *S. ixodetis*, or *S. mirum* with or without antibiotics (penicillin G sodium salt). Out of 70 ticks used, a total of 63 ticks were alive after the injection. Seven ticks were dead during the incubation period seven days after injection. The average engorgement weight was calculated for 3–7 individuals in each group and fell in the range of 87.4–262.2 mg. The largest engorgement weight was observed for the *S. ixodetis* (5 × 10^11^ bacteria) + penicillin G sodium salt injection group (Fig. 1, *S. ixodetis* H_PG). Meanwhile, the smallest was for the *S. mirum* (5 × 10^11^ bacteria) injection group (Fig. 1, *S. mirum* H_wo). There were no statistically significant differences in engorgement weights between the groups, including those with and without antibiotics, by the Kruskal–Wallis test (Fig. 1). The egg production efficiency was calculated as the laid egg weight divided by the engorged body weight^31^. The average calculated for 3–7 individuals in each group fell in the range of 33.1–57.1. The largest egg production efficiency was observed in the *S. mirum* (5 × 10^8^ bacteria) injection group (Table 1, SM_Low_wo), whereas the smallest was observed in the *S. ixodetis* (5 × 10^11^ bacteria) injection group (Table 1, SI_High_wo). There were no statistically significant differences in the egg production efficiency between the groups, including those with and without antibiotics. The hatching rate was calculated for 3–7 individuals in each group and fell in the range of 3.5–98.8%. The highest median hatching rate was observed for the *S. ixodetis* (5 × 10^8^ bacteria) injection group (Table 1, SI_Low_wo). Meanwhile, the lowest was observed for the *S. mirum* (5 × 10^11^ bacteria) + penicillin G sodium salt injection group (Table 1, SM_High_PG). There were no statistically significant differences in the hatching rate among the groups, including those with and without antibiotics (Fig. 1, Table 1).

**Table 1:** The egg weight, rate of egg hatching, and detection of *Spiroplasma* from eggs and larvae of *Haemaphysalis longicornis* according to injection group.

Group	Mean of Egg weight/body weight (SD)	Median of hatching rate (IQR) (%)	Detection of *Spiroplasma* from egg (No. of positive)	Detection of *Spiroplasma* from larvae (No. of positive)	Sample size (No. of fed females)
PBS	45.1 (6.5)	89.9 (72.5–95.2)	0	0	6
PBS_PG	44.9 (5.3)	88.6 (73.3–94.7)	0	0	7
SI_High_wo	38.3 (5.4)	80.3 (48.4–84.8)	2 (three pools each)	2 (three pools each)	7
SI_High_PG	46.9 (1.8)	84.0 (78.4–97.3)	1 (two pools)	1 (two pools)	3
SI_Low_wo	45.9 (6.0)	92.7 (56.1–95.1)	0	0	7
SI_Low_PG	46.6 (1.6)	80.9 (64.6–95.7)	0	0	6
SM_High_wo	49.2 (3.6)	83.8 (58.9–88.6)	0	0	7
SM_High_PG	39.3 (12.7)	65.6 (31.8–82.5)	0	0	7
SM_Low_wo	51.1 (3.4)	83.6 (77.1–85.3)	0	0	7
SM_Low_PG	50.3 (4.7)	73.7 (47.6–91.2)	0	0	6

PBS_PG, PBS + penicillin G sodium salt; SI_High_wo, *S. ixodetis* (5 × 10^11^ bacteria); SI_High_PG, *S. ixodetis* (5 × 10^11^ bacteria) + penicillin G sodium salt; SI_Low_wo, *S. ixodetis* (5 × 10^8^ bacteria); SI_Low_PG, *S. ixodetis* (5 × 10^8^ bacteria) + penicillin G sodium salt; SM_High_wo, *S. mirum* (5 × 10^11^ bacteria); SM_High_PG, *S. mirum* (5 × 10^11^ bacteria) + penicillin G sodium salt; SM_Low_wo, *S. mirum* (5 × 10^8^ bacteria); SM_Low_PG, *S. mirum* (5 × 10^8^ bacteria) + penicillin G sodium salt; SD, standard deviation; IQR, interquartile range.

### Detection of *Spiroplasma* in eggs and larvae from experimentally inoculated *H. longicornis*

The presence of *Spiroplasma* in the eggs was tested by PCR using three egg pools per tick. In total, egg pools from two ticks comprising three pools each in the *S. ixodetis* (5 × 10^11^ bacteria) injection group and one tick (two pools) in the *S. ixodetis* (5 × 10^11^ bacteria) + penicillin G sodium salt injection group were positive for *Spiroplasma* infection (Table 1). The rest of the groups tested negative by *Spiroplasma*-specific PCR. No *Spiroplasma* was detected in any of the *S. mirum* injection groups.

The presence of *Spiroplasma* in the larvae was tested using PCR with three larval pools per individual tick. In total, larvae pools from two ticks comprising three pools each in the *S. ixodetis* (5 × 10^11^ bacteria) injection group and one tick (two pools) in the *S. ixodetis* (5 × 10^11^ bacteria) + penicillin G sodium salt injection group were positive for *Spiroplasma* infection (Table 1). The rest of the groups were all negative by *Spiroplasma*-specific PCR. No *Spiroplasma* was detected in any of the *S. mirum* injection groups.

## Discussion

*Spiroplasma* has been identified as one of the core microbial taxa in the tick microbiome^25^. However, its transmission dynamics in nature remains unknown in ticks. This study used field-collected *I. ovatus*, a species harbouring *Spiroplasma* at high frequencies, for experimental infestation under laboratory settings. It was confirmed that *Spiroplasma* is transmitted to eggs and larvae. This study has provided the first experimental evidence that *S. ixodetis* is vertically transmitted in ticks.

Previously, it was suggested that horizontal transmission of *Spiroplasma* between mites and *Drosophila* (*Drosophila nebulosa* and *Drosophila willistoni*) could occur under experimental conditions^32^. *Spiroplasma ixodetis* has been detected in several arthropods, including some species of ticks. Phylogenetic analysis has suggested horizontal transmission between ticks and other arthropods^24^. In this study, *S. ixodetis* and *S. mirum* were experimentally inoculated into *Spiroplasma*-free *H. longicornis*. *Spiroplasma* was detected only in the *S. ixodetis* injection groups, but not in the *S. mirum* injection groups (Table 1). This result provides the first experimental evidence that *S. ixodetis* can be maintained in the non-native tick species, supporting the horizontal transmission potential of *Spiroplasma* among different tick species. Having said that, the lower frequency of *Spiroplasma* transmission may have resulted from the poor adaptation of the isolates to the new host tick species. *Spiroplasma* strains introduced into other species are often poorly transmitted from the mother to offspring in *Drosophila*^33^. *Spiroplasma citri*, which normally infects leafhoppers, grows well in *D. melanogaster* haemolymph but cannot access the oocyte and, therefore, is not vertically transmitted^34^. Although a number of interspecific transfers of *Spiroplasma* between *Drosophila* species have been confirmed^35^, the transfer of *Spiroplasma* from *Drosophila* to other arthropod species has rarely been documented. The vertical transmission rate of *Spiroplasma* in *Drosophila* was found to be severely affected by temperature^36^, indicating that environmental factors under laboratory settings, such as temperature, may have affected the results.

*Spiroplasma poulsonii* in *Drosophila* colonises host oocytes at specific stages, coinciding with vitellogenesis, and requires yolk transport and uptake machinery to achieve efficient vertical transmission^22^. *Spiroplasma citri* has been observed entering the salivary cells of the beet leafhopper by receptor-mediated endocytosis^37^. These findings suggest that *Spiroplasma* may have a general capacity to interact with the host endocytic machinery to ensure vertical transmission. Vitellogenin uptake through the vitellogenin receptor in the oocyte is an essential event for the progress of oogenesis in ticks, including *H. longicornis*^38,39^. Therefore, *Spiroplasma* in ticks may use the same mechanism as that observed in other arthropods. To further confirm this hypothesis, the localization of *S. ixodetis* in *I. ovatus* after the blood feeding up to oviposition needs to be clarified.

In this study, *Spiroplasma* was inoculated with antibiotics in several injection groups. However, no significant difference was observed in terms of engorged weight, egg weight, and hatching rate between the antibiotic-treated and non-treated groups. When the symbiont density in host insects has been experimentally decreased using antibiotics, host reproduction ability is reduced because of symbiont sorting upon vertical transmission^40^. In the present study, antibiotic treatment had no effect on the vertical transmission of *Spiroplasma* in ticks. Antibiotic treatment for ticks has been associated with several reproductive dysfunctions, such as reduced engorged and egg weight and hatching rate. This is because of dysbiosis of microbiota and reduction of endosymbionts^41,42^. In our preliminary experiments, ticks injected with 0.5 units of penicillin G sodium salt died within 24 h, indicating an adverse effect of antibiotics on tick physiology (data not shown). The lack of a pronounced effect from the antibiotic treatment in the current experiment may be partially explained by the lower concentration of antibiotics where the microbiota was not affected in the antibiotic-treated groups. Therefore, it is necessary to optimise the concentration and type of antibiotics to be administered as well as inoculation routes in the future to understand the interaction between *Spiroplasma* and tick microbiota.

## Materials and methods

### Ticks

*Ixodes ovatus* were collected by flagging the vegetation in Sapporo city, Japan (N 43.02 E 141.29) in May 2021 and identified morphologically under a stereomicroscope according to the standard morphological keys^43^. Field-collected ticks were transferred to Petri dishes and preserved in an incubator at 16 °C until use. Parthenogenetic laboratory colonies of *H. longicornis* Okayama strain (Fujisaki, 1978)^44^, maintained at the National Research Center for Protozoan Diseases, Obihiro University of Agriculture and Veterinary Medicine, Japan, were used in *Spiroplasma* infection experiments. This tick colony was confirmed to be *Spiroplasma*-free by *Spiroplasma*-specific PCR prior to the experiment.

### Experimental infestation of field-collected *Ixodes ovatus* on rabbits

**Figure 2:**
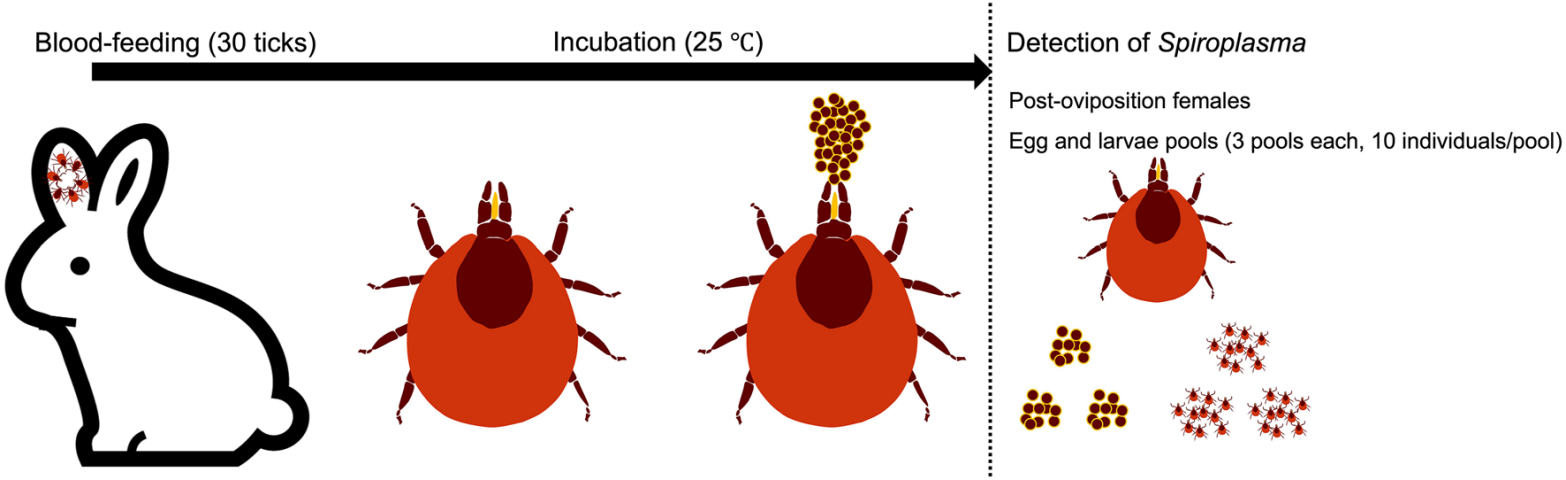
Overview of experimental flow of experimental infestation of field-collected *Ixodes ovatus* on rabbits.

Female and male ticks were placed in the same Petri dish a week before the experiment. Thirty female *I. ovatus* were attached to the ears of Japanese white rabbits (Slc: JW/CSK; Japan SLC, Shizuoka, Japan) using earbags. The attached ticks were allowed to feed until they became engorged and detached naturally. The fully engorged ticks were then collected and incubated in the dark at 25 °C with saturated humidity for oviposition (Fig. 2).

The pools of eggs (*n* = 10) and larvae (*n* = 10) were collected from each tick and subjected to DNA extraction and *Spiroplasma*-specific PCR, as described below.

### *Spiroplasma* isolates

In this study, two species of *Spiroplasma*: *S. ixodetis* (strain 135) and *S. mirum* (strain Q35) were used. *Spiroplasma ixodetis* strain 135 was isolated from male *Ixodes monospinosus* using ISE6 cells in a previous study^45^. The isolate was grown in ISE6 cells received from the CEH Institute of Virology and Environmental Microbiology (Oxford, UK) with L-15B medium supplemented with 10% foetal bovine serum and 5% tryptose phosphate broth (Sigma-Aldrich, St. Louis, MO, USA) at 32 °C as described previously^30^ . *Spiroplasma mirum* strain Q35 was isolated from a female *Ixodes pavlovsky* collected at Urausu town, Japan (N 43.46, E 141.76) by co-culturing the tick homogenate with ISE6 cells. The bacterial species was identified by amplifying and sequencing the 16S rDNA sequence as previously reported (unpublished)^45^. The isolate was thereafter cultured using modified SP4 medium at 32 °C^46^. The culture medium was changed when the colour changed from red to yellow. The titration of *Spiroplasma* was conducted by counting the bacterial cells under a dark-field microscope. The final concentrations of *Spiroplasma* solution were adjusted to 1 × 10^9^ and 1 × 10^12^ bacteria/µL using culture media prior to inoculation into the ticks.

### Experimental infestation of *Spiroplasma*-inoculated *Haemaphysalis longicornis* on rabbits

Females of *H. longicornis* were attached to glass slides and injected with 0.5 µL of PBS or *Spiroplasma* solution through the fourth coxae using an IM 300 Microinjector (Narishige, Tokyo, Japan). There were 10 injection groups, namely the PBS groups, PBS only, and PBS + penicillin G sodium salt (Sigma-Aldrich, St. Louis, MO, USA); *S. ixodetis* injection groups, *S. ixodetis* (5 × 10^8^ bacteria and 5 × 10^11^ bacteria) only and *S. ixodetis* (5 × 10^8^ bacteria and 5 × 10^11^ bacteria) + penicillin G sodium salt; *S. mirum* injection groups, *S. mirum* (5 × 10^8^ bacteria and 5 × 10^11^ bacteria) only and *S. mirum* (5 × 10^8^ bacteria and 5 × 10^11^ bacteria) + penicillin G sodium salt. The final concentration of penicillin G sodium salt in the solution was 100 U/mL.

**Figure 3:**
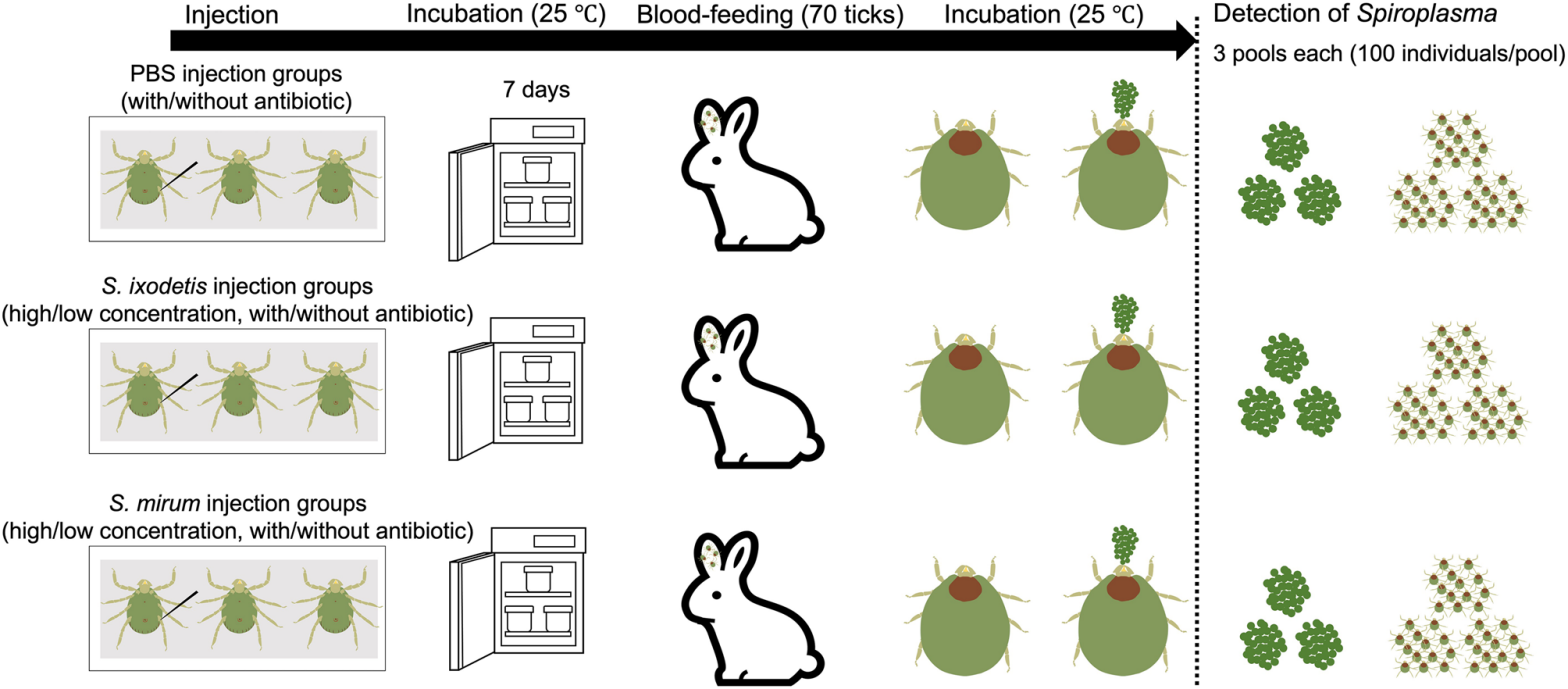
Overview of experimental flow of *Spiroplasma*-injection into *Haemaphysalis longicornis* ticks and their infestation on rabbits.

After inoculation, the ticks injected were left for seven days at 25 °C in an incubator. For rabbit infestation, a total of 70 ticks per injection group were attached to separate ears of Japanese white rabbits. The attached ticks were allowed to feed until they became engorged and detached naturally. The fully engorged ticks were then weighed and incubated in the dark at 25 °C with saturated humidity for oviposition. After oviposition, the eggs were weighed and incubated under the same conditions until hatching. The hatching rate was calculated by counting the number of hatched larvae among the selected pools of eggs (50–150 eggs/pool). Three pools of eggs (100 each) and larvae (100 each) from each tick were subjected to DNA extraction and *Spiroplasma*-specific PCR (Fig. 3).

### DNA extraction and *Spiroplasma*-specific PCR

The eggs, larvae, and post-oviposition females were homogenised using a BioMasher (Nippi, Tokyo, Japan), as described in the manufacturer’s protocol. Genomic DNA was extracted from the homogenates using a NucleoSpin® DNA Insect Kit (Macherey–Nagel GmbH & Co. KG, Düren, Germany), following the manufacturer’s guidelines. To detect *Spiroplasma* DNA, PCR amplification targeting 1028 bp of 16S rDNA was performed using the primers, spi_f1 (5′-GGGTGAGTAACACGTATCT-3′) and spi_r3 (5′-CCTTCCTCTAGCTTACACTA-3′)^30^. PCR was conducted in a 20 μL reaction mixture containing 10 μL of 2 × Gflex PCR Buffer (Mg^2+^, dNTP plus) (TaKaRa Bio Inc., Shiga, Japan), 400 nM of Tks Gflex™ DNA Polymerase, 400 nM of each primer, 1 μL of DNA template, and sterilised water. The reaction was performed at 94 °C for 1 min, followed by 45 cycles at 98 °C for 10 s, 60 °C for 30 s, and 68 °C for 45 s, and a final step at 68 °C for 5 min. PCR products were electrophoresed on a 1.0% agarose gel. The DNA of *Spiroplasma* species isolated from *I. persulcatus* in the previous study^47^ and sterilised water were included in each PCR run as positive and negative controls, respectively. The amplified PCR products were purified using the ExoSAP-IT Express PCR Cleanup Reagent (Thermo Fisher Scientific, Tokyo, Japan). Sanger sequencing was performed using a BigDye Terminator version 3.1 Cycle Sequencing Kit (Applied Biosystems, Foster City, CA, USA). Sequencing data were assembled using the ATGC software version 6.0.4 (GENETYX, Tokyo, Japan).

### Statistical analyses

All the statistical analyses were performed using Microsoft 365 Excel. The Kruskal–Wallis test was used to confirm significant differences in engorged tick weight, egg weight, and egg hatching rate per injection group. R software (version 4.2.3) was used to create the graphs^48^.

### Ethical statement

All the animal experiments were carried out under the guidance of the Institute for Laboratory Animal Research (ILAR), which was based on Fundamental Guidelines for Proper Conduct of Animal Experiment and Related Activities in Academic Research Institutions under the jurisdiction of the Ministry of Education, Culture, Sports, Science and Technology, Japan. This experimental protocol was approved by the Committee on the Ethics of Animal Experiments of Hokkaido University (Approval No. 22-0030) and the Animal Care and Use Committee of Obihiro University of Agriculture and Veterinary Medicine (Approval Nos. 18-11, 19-74, 20-85, and 21-40). All experiments were performed in accordance with the guidelines of the committee. The study is reported in compliance with the ARRIVE guidelines.

## Conclusion

This study is the first to experimentally confirm the vertical transmission of *S. ixodetis* in ticks using field-collected *I. ovatus*. In addition, *Spiroplasma* was detected in eggs and larvae originating from *H. longicornis* experimentally inoculated with *S. ixodetis*. This has indicated that *S. ixodetis* can be transferred into the non-native arthropod species via horizontal transmission. Further experiments are needed to evaluate the viability of the introduced *Spiroplasma* in the recipient hosts and the transmission efficacy to the next developmental stage or generation. These findings may help understand the transmission dynamics of *Spiroplasma* in nature, the adaptation mechanism to specific tick species, and ultimately the effects on host tick physiology.

## Data Availability

The datasets used and/or analysed during the current study available from the corresponding author on reasonable request.
